# Activated Monocytes Enhance Platelet-Driven Contraction of Blood Clots via Tissue Factor Expression

**DOI:** 10.1038/s41598-017-05601-9

**Published:** 2017-07-11

**Authors:** Alina D. Peshkova, Giang Le Minh, Valerie Tutwiler, Izabella A. Andrianova, John W. Weisel, Rustem I. Litvinov

**Affiliations:** 1 Institute of Fundamental Medicine and Biology, Kazan Federal University, Kazan, 420012 Russian Federation; 2 Department of Cell and Developmental Biology, University of Pennsylvania Perelman School of Medicine, Philadelphia, Pennsylvania 19104 USA; 3 School of Biomedical Engineering, Sciences and Health Systems, Drexel University, Philadelphia, Pennsylvania 19104 USA

## Abstract

Platelet-driven reduction in blood clot volume (clot contraction or retraction) has been implicated to play a role in hemostasis and thrombosis. Although these processes are often linked with inflammation, the role of inflammatory cells in contraction of blood clots and thrombi has not been investigated. The aim of this work was to study the influence of activated monocytes on clot contraction. The effects of monocytes were evaluated using a quantitative optical tracking methodology to follow volume changes in a blood clot formed *in vitro*. When a physiologically relevant number of isolated human monocytes pre-activated with phorbol-12-myristate-13-acetate (PMA) were added back into whole blood, the extent and rate of clot contraction were increased compared to addition of non-activated cells. Inhibition of tissue factor expression or its inactivation on the surface of PMA-treated monocytes reduced the extent and rate of clot contraction back to control levels with non-activated monocytes. On the contrary, addition of tissue factor enhanced clot contraction, mimicking the effects of tissue factor expressed on the activated monocytes. These data suggest that the inflammatory cells through their expression of tissue factor can directly affect hemostasis and thrombosis by modulating the size and density of intra- and extravascular clots and thrombi.

## Introduction

Clot contraction or retraction is the volumetric shrinkage of blood clots^1, 2^, and is driven by contractile forces generated by platelets^1, 3^. This process, which occurs *in vitro* and *in vivo*, has been implicated in the restoration of blood flow past otherwise obstructive thrombi and has the potential to be a vital compensatory mechanism in thrombotic states^1, 4–6^. The cellular composition of the blood has been found to influence the rate and extent of clot contraction^7^, however, the role of activated monocytes in clot contraction has not been investigated. Leukocytes, such as monocytes and neutrophils, are recruited to the site of vessel injury and present in increased numbers in patients with various thrombotic disorders^8^.

Local inflammation can enhance hemostatic processes by amplifying clot initiation and limiting fibrinolysis^9^. In particular, inflammatory cells such as monocytes are known to express tissue factor (TF) on their surfaces in various disease states such as atherosclerosis, heart disease, and severe sepsis^10–12^. TF expression is known to occur following activation with agonists such as phorbol-12-myristate-13-acetate (PMA) and bacterial lipopolysaccharide^13, 14^. TF on activated blood and endothelial cells, through its role in thrombin formation, is critical for clot formation and clot contraction to occur^1, 15^. Thrombin converts fibrinogen to fibrin^16^ and activates platelets^17^, which facilitates changes needed for platelet actin-myosin contraction to occur^18^.

Blood cells and vascular endothelial cells that come in direct contact with the circulating blood do not express TF under normal physiological conditions. TF is expressed by endothelial and mononuclear cells in response to injury, as well as to a number of different inflammatory extracellular stimuli, including LPS, TFN-a, IL-1, IL-2, IL-6, interferon-γ, as well as in response to initiation of the coagulation process and thrombosis^19, 20^. TF produced by monocytes is the main source of blood-borne TF^19, 20^.

The expression of TF on monocytes is involved in thrombus formation in various pathological conditions such as inflammatory diseases^21^, sepsis^22^, and cancer^23^. Recently, monocyte-expressed TF has been proposed as the main protein responsible for the thrombotic complications of atherosclerosis. The presence of TF coding mRNA has been reported in macrophage foam cells and monocytes adjacent to the cholesterol clefts in atherosclerotic plaques from patients undergoing carotid endarterectomy^24^. A role for monocyte TF has been proposed in unstable coronary syndromes^11, 25^. In particular, increased expression of TF was found in atherectomy specimens from patients with unstable angina^25, 26^ or myocardial infarction^27^.

Clinical studies have confirmed that activated monocytes contribute to thrombosis mainly because of their TF expression^28, 29^. After plaque injury, as is observed in angioplasty, exposure of cellular and extracellular TF to the circulating blood plays a pivotal role in mediating fibrin-rich thrombus formation leading to acute coronary syndromes^30^. TF expressed on monocytes/macrophages is up-regulated by inflammatory cytokines and oxidized lipids from the plaque^31^. Despite ample evidence for the critical importance of monocyte-expressed TF in thrombosis, the role of inflammatory cells bearing TF in the remodeling of blood clots has never been studied. The novelty of our work is the effects of activated monocytes mediated by TF on clot contraction.

Collectively, these data motivate a need to test the role of activated monocytes as modulators of clot contraction, which may be a pathogenic factor that regulates local blood flow and potentially affects the course and outcomes of ischemic tissue damage. In addition, modulating the degree of clot shrinkage or densification may affect permeability and hence the susceptibility of a clot or thrombus to enzymatic lysis, and make it more or less stable and/or prone to embolization. We hypothesize that activated monocytes enhance the process of clot contraction, which may suggest an additional pathophysiologic mechanism of shrinkage of a hemostatic clot or obstructive thrombus in inflammatory conditions with important pathogenic consequences.

## Results

### Activated monocytes stimulate clot contraction

**Figure 1:**
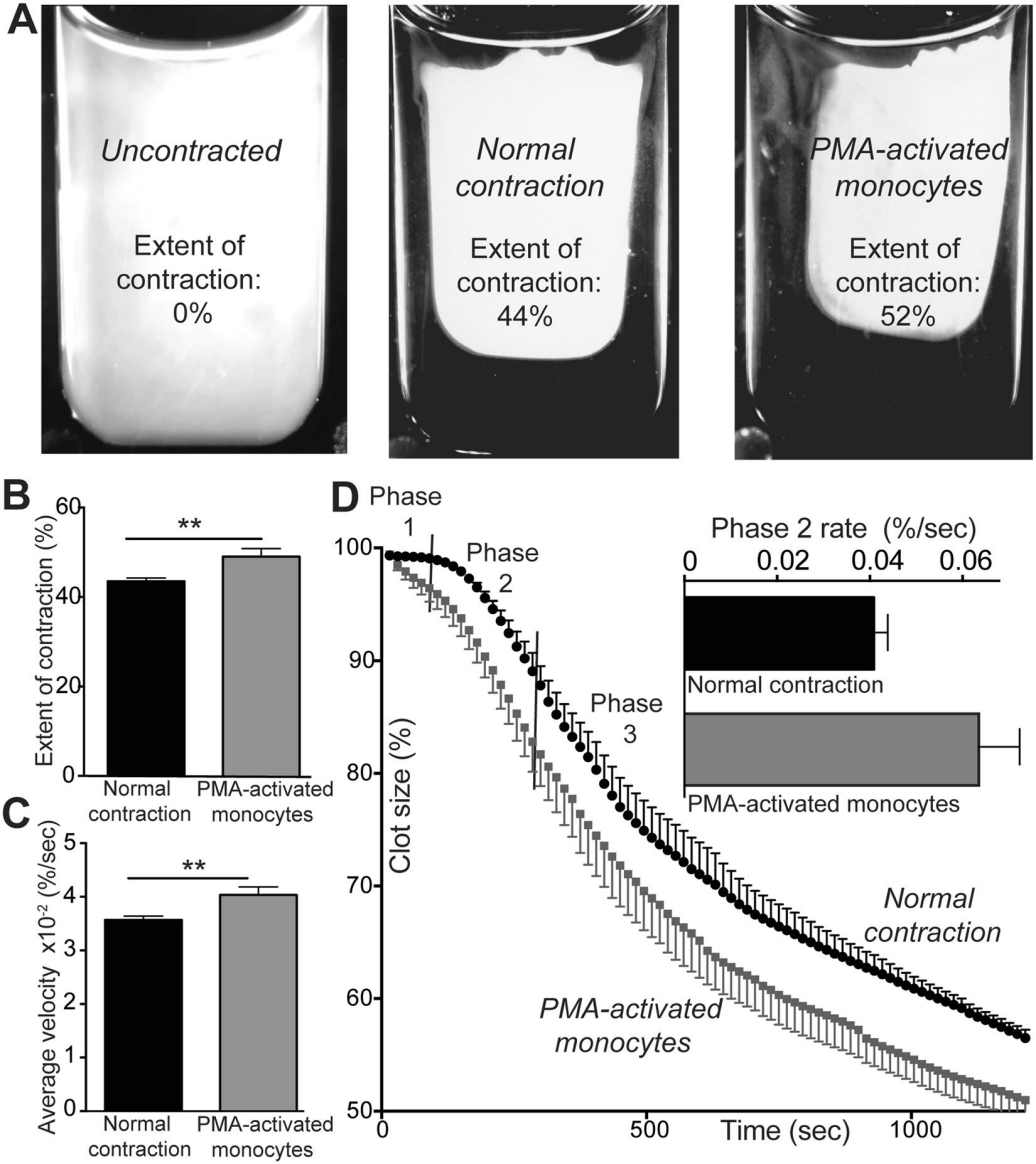
Effects of activated monocytes on clot contraction. Isolated non-activated monocytes or PMA-activated monocytes were supplemented to whole citrated blood. Clotting and clot contraction were initiated with 2 mM CaCl_2_ and 1 U/ml thrombin followed by optical tracking of clot size. (**A)** Representative raw images showing an initially uncontracted clot and clots after 20-minute contraction obtained from the same blood sample under standard experimental conditions after addition of non-activated and PMA-activated monocytes. (**B**) Average extent of clot contraction and (**C**) average velocity of contraction at 20 minutes, determined after addition of non-activated and PMA-activated monocytes (n = 8). (**D**) The averaged kinetic contraction curves (n = 8) with and without monocyte activation were used to determine the rates of the three phases of clot contraction separated by the vertical lines. *Insert* – the comparative rates of Phase 2. The results are presented as mean ± SEM. **p < 0.01.

Since monocytes are incorporated into clots and thrombi, we hypothesized that monocytes have the potential to influence the rate and extent of clot contraction by direct or indirect cooperation with platelets^32^. PMA-activated monocytes added to whole blood were found to enhance the extent and velocity of clot contraction (Fig. 1A–C) when compared to monocytes that had not been activated with PMA. Addition of non-activated monocytes alone did not change contraction parameters, such that under the same conditions the extent of contraction with and without resting monocytes was 51 ± 3% and 48 ± 1%, respectively (p = 0.3).

**Figure 3:**
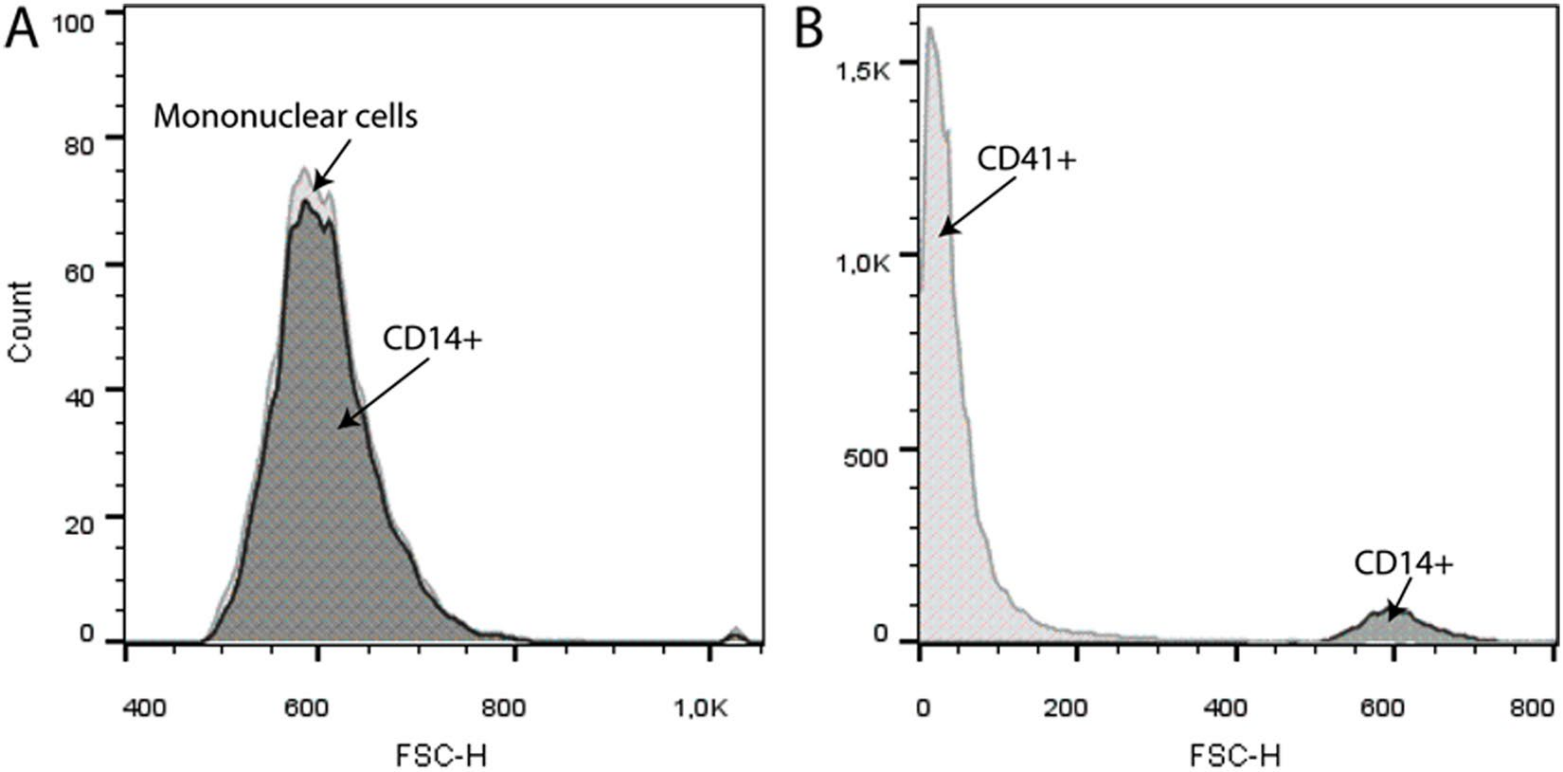
Flow cytometry of isolated monocytes. Representative raw data of a flow cytometry experiment with a freshly isolated monocyte preparation incubated with mouse anti-human CD14 antibodies (monocytes are CD14-positive) and mouse anti-human CD41 antibodies (platelets are CD41-positive) for 30 min at 4 °C in the dark. The preparation contains ~98% CD14+ monocytes ((**A**) dark grey) of all mononuclear cells ((**A**) light grey). The ratio of CD14+ monocytes ((**B**) dark grey) to CD41+ platelets ((**B**) light grey) is roughly 1:10.

To exclude the possibility that the observed effects were due to contaminating platelets, we performed control experiments with isolated gel-filtered platelets activated with PMA (Fig. S1). We added them to the blood samples in a corresponding amount, which comprised ~1–2% of the platelet count naturally present in the blood sample. Under these conditions, activated platelets by themselves did not induce any noticeable increase in the extent of clot contraction, which was 47 ± 4% (PMA-activated platelets) vs 51 ± 1% (non-activated platelets, p = 0.4), indicating that the detected effects were due to the activated monocytes that augmented the contractile activity of endogenously present platelets. Monocyte preparations contained 98–100% monocytes of all mononuclear cells and the contamination of platelets varied from 10 to 27 platelets/monocyte (Fig. 3).

### Phase analysis

The results of our previous study^7^ show that clot contraction is a dynamic process that is best described as following three kinetically discernable phases that result in reducing the bulk volume of the clot over time: an initiation period (Phase 1), which is followed by “linear contraction” (Phase 2) and then clot stabilization (Phase 3). Specifically, Phase 1 requires platelets and fibrinogen, Phase 2 requires the additional presence of RBCs, and Phase 3 requires cross-linking of fibrin by factor XIIIa. Here, we found that Phase 1 and Phase 3 had the same rate constants in both the presence and absence of activated monocytes (Fig. 1D), while Phase 2 was characterized by an increase in the rate of contraction (Fig. 1D - insert), suggesting that the PMA-stimulated monocytes assist the actively contracting platelets in better overcoming the resistance to contraction of the erythrocyte/fibrin matrix.

It has been recently shown that the mechanical properties of the constitutive components of the clot and their dynamic interactions largely determine the kinetics and the degree of clot contraction^7, 33^. Platelets have an active contractile machinery that generates mechanical forces propagated across the clot. Fibrin is a viscoelastic material that can be described mechanically as a semiflexible and deformable polymer network. The incorporation of RBCs in the blood clot volume modulates the uniformity of the fibrin network, affects the mechanical properties of the clot, and impedes the process of clot contraction proportionally to the volume fraction of RBCs^7^. RBCs are easily deformed and when subjected to compressive forces such as those generated by contracting platelets the RBCs become packed in the core of the blood clot and take on a polyhedral shape^4^. Based on the quantitative prediction of a recently developed model of clot contraction^33^, the monocytes added to the blood by themselves will not have much of an effect on the mechanics of clot contraction because the volume fraction and the number of exogenous and endogenous monocytes [about (0.5–1.0) × 10^6^ cells/ml] is miniscule compared to the RBCs and platelets.

### The promoting effect of activated monocytes on clot contraction is mediated by TF expression

**Figure 2:**
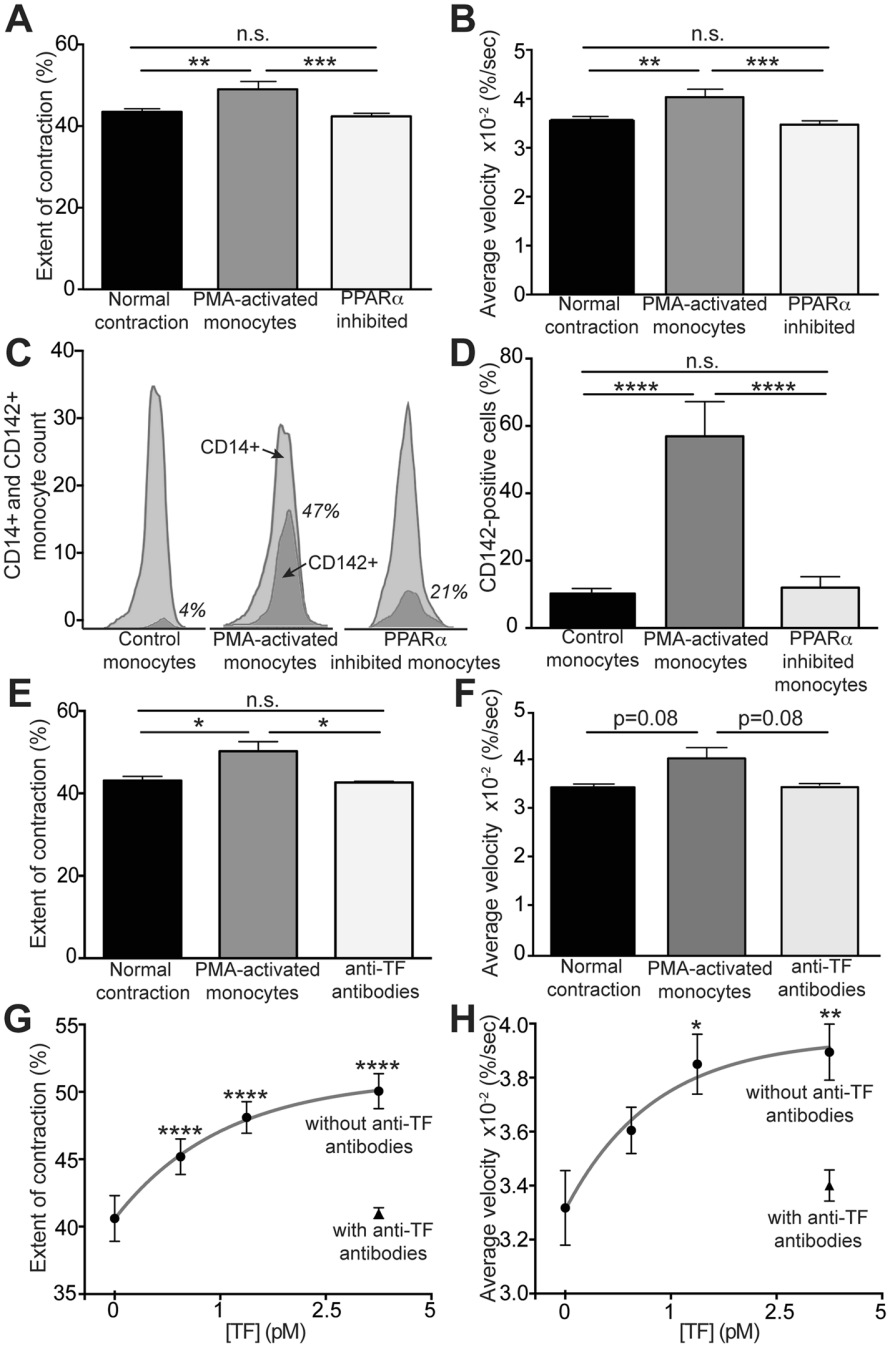
Effects of cell-expressed or purified TF on clot contraction. (**A**) Average extent of clot contraction and (**B**) average velocity of clot contraction at 20 minutes after addition to whole blood of non-activated monocytes (normal contraction), PMA-activated monocytes, and PMA-activated monocytes treated with a PPARα agonist, an inhibitor of TF expression, prior to PMA activation (n = 24). (**C**,**D**) The fraction of TF-expressing (CD142-positive) monocytes determined by flow cytometry in the CD14-positive monocyte populations under various experimental conditions indicated. (**C**) Raw flow cytometry peaks from a representative experiment are shown. The monocyte population is shown in light gray peaks, while the fractions of TF-expressing monocytes are shown in dark gray peaks and numbers in *italics*. (**D**) The average percent of TF-expressing monocytes under various experimental conditions indicated (n = 3). (**E**) Average extent of clot contraction and (**F**) average velocity of clot contraction at 20 minutes after addition to whole blood of non-activated monocytes (normal contraction), PMA-activated monocytes and PMA-activated monocytes treated with inhibitory anti-TF antibodies (n = 3). **(G)** Average extent of clot contraction and **(H)** average velocity of clot contraction at 20 minutes after addition to whole blood of lipidated TF at a final concentration of 1 pM, 2.5 pM, and 5 pM fitted with an exponential (n = 4). The results are presented as mean ± SEM. *p < 0.05, **p < 0.01, ***p < 0.001, ****p < 0.0001.

In certain pathological conditions, activated monocytes can express TF, a potent initiator of clotting via the extrinsic pathway, on their cell surface^10–12^. To probe whether TF expression is the mechanism through which activated monocytes influence the process of clot contraction, we suppressed the TF activity in two ways. First, we applied a PPARα agonist, which inhibits signaling pathways involved in TF expression^34, 35^. The addition of PPARα agonist-treated monocytes resulted in a restoration of clot contraction parameters to being insignificantly different from the control (with or without non-activated monocytes) and significantly different from activated monocytes (Fig. 2A,B). Importantly, this compound caused a 78% decrease in the average number of monocytes bearing TF on their surface following 15 minutes of incubation with PMA (Fig. 2C,D). Second, since the PPARα agonist may have other effects on monocyte signaling pathways, anti-TF antibodies were used to selectively inhibit procoagulant TF activity. Treatment of PMA-activated cells with anti-TF antibodies completely abrogated the stimulating effect of activated monocytes on the extent of clot contraction down to the level of non-activated monocytes (Fig. 2E,F). This result corroborates the conclusion that PMA-activated monocytes enhance clot contraction through their TF expression.

Conversely, the addition of lipidated purified TF to whole blood was used to mimic the TF expression on PMA-activated exogenous monocytes added to blood samples. TF caused a dose-dependent exponential increase in the extent and rate of clot contraction (Fig. 2G,H), which was abrogated in the presence of anti-TF antibodies (Fig. 2G,H), the same used to suppress TF on the PMA-activated monocytes (Fig. 2D).

In summary, our data provide evidence that the mechanism by which activated monocytes assist the contracting platelets in overcoming the resistance of the RBC/fibrin matrix is due to enhanced platelet activation via additional endogenous thrombin generated by monocyte-expressed TF. The stimulating effects of activated monocytes on clot contraction were eliminated by inhibition of TF expression and were reproduced by addition of purified TF.

## Discussion

**Figure 4:**
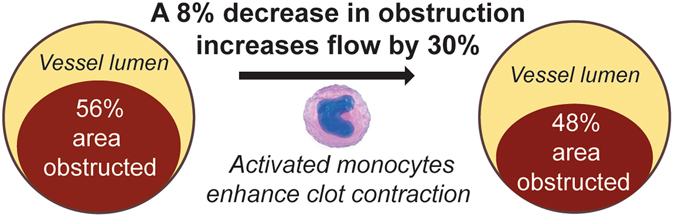
Relationship between the degree of vessel obstruction and blood flow. Thrombotic vessel obstruction increases the vascular resistance as described by Poiseuille’s law, which says that resistance is inversely related to the radius to the fourth power. Therefore, if the radius (or diameter) of a vascular segment is reduced by one-half, the resistance or the volumetric blood flow within that narrowed segment increases by 16-fold, which is a dramatic effect relative to the change in degree of vessel obstruction. Based on the Poiseuille’s equation: *Volume flow rate* = π*(*pressure difference*)*(*radius*)4/8*(*viscosity*)*(*length*) the change in the cross-sectional area corresponding to a decrease of the degree of vessel obstruction from 56% to 48% will result in an increase in the volumetric blood flow by about 30%.

The degree of contraction can be an important modulator of the blood flow in thrombotic conditions and there is emerging indirect evidence that the degree and the rate of contraction of clots and thrombi may be an important pathogenic factor that affects the local blood hydrodynamics and the course and outcome of thrombosis^5, 6, 36^. Although the severity of thrombosis and thromboembolism is largely determined by the diameter and location of the occluded vessel, the ability of the thrombi to contract either more or less can dramatically affect the degree of vessel obstruction and its hydrodynamic consequences. Based on Poiseuille’s Law, if thrombosis results in an 80% reduction in the vessel cross-sectional area, this would translate to a reduction of blood flow to 4% of that with no clot. Figure 4 shows that relatively minor changes in the cross-sectional area of a thrombus (by about 8% as observed in our experiments) cause significant changes in the volumetric blood flow (by about 30%). Moreover, there is a strong inverse correlation between the degree of arterial blockage and flow shear rate, which is a strong modulator of platelet function and thrombus formation, growth and stability. Therefore, even a relatively small variation in the degree of contraction of an obstructive thrombus or thrombotic embolus, can cause substantial hemodynamic and pathogenic consequences. Our results indicate that activated inflammatory cells can strongly modulate the local blood flow in the obstructed vessels by changing the size of a thrombus formed at the site of inflammation.

The promoting effect of activated monocytes on clot contraction is mediated by TF on the cell surface. Regardless of whether it is expressed on cells or purified, TF leads to thrombin production, which triggers platelet activation and contraction. Based on the total content of TF in PMA-activated monocytes of about 100 pg/10^6^ cells (as determined by ELISA of the cell lysates), the amount of TF added to the blood with ~0.3 × 10^6^/ml PMA-activated monocytes in our experiments is ~30 pg/ml or ~1 pM (with TF mol. weight of 35 kDa), which is enough for intensive thrombin generation^37^. As determined with ELISA of the commercially available stock solution of TF (EXTEM), the final concentrations of TF added to the blood in our experiments corresponds to 5, 2.5, and 1.1 pM of TF, respectively, which is similar to the amount added with PMA-activated monocytes. It has been shown that activated monocytes at a concentration of 0.5 × 10^6^/ml generate ~200 nM thrombin^38^, then in our experiments the PMA-activated monocytes added at 0.3 × 10^6^/ml should have generated ~120 nM of endogenous thrombin, which is more than 8 nM of exogenous thrombin (see calculations in the Supplementary Information). This amount of endogenous thrombin is still below an overall thrombin generation potential determined by the normal prothrombin level of about 1.3 µM in plasma or 700 nM in whole blood^39^.

It is not unexpected that PMA-activated monocytes enhance clot contraction; however, the TF-dependent enhancement of clot contraction occurs on top of the high activity of exogenous thrombin added to induce clot formation and platelet activation. This points to an underappreciated importance of endogenously or locally generated thrombin activity for the process of clot contraction. It suggests that endogenous thrombin generated at the site of thrombosis is a more potent platelet activator due to higher local enzymatic activity, resistance to antithrombins, and/or preferential localization on or near the platelet membrane.

It is possible that the exogenous thrombin, like thrombin formed in the systemic blood flow, is inactivated quickly, while the endogenously or locally generated thrombin is being formed continuously over time after the overall antithrombin potential has been reduced or exhausted. From the literature, the relative sensitivity of systemic versus local thrombin activity to antithrombins has been studied in clinical and experimental settings. It has been shown that acute coronary syndrome patients did not benefit when given systemic direct antithrombins in a safe dose, implying that local thrombin-induced platelet activation is prevalent and less sensitive to thrombin inhibition^40^. Similarly, inhibition of the coagulation factors X, XI or thrombin dramatically reduce the degree of platelet activation and microaggregate formation in the bloodstream without affecting the degree of local platelet deposition and aggregation on the surface of immobilized collagen^41^. These and other data support the notion that endogenous thrombin generated at the site of thrombosis is a more potent platelet activator for a number of reasons, including resistance to antithrombins.

Despite a large gap in the fundamental understanding of mechanisms that control clot shrinkage *in vitro* and *in vivo*, there are a number of conceivable mechanisms that underlie the potential effects of clot contraction on the course of ischemic tissue damage, e.g. in ischemic stroke. Obviously, a thrombus or thrombotic embolus results in blockage of blood flow to a portion of the brain; size and location of the thrombotic event influences stroke severity and functional outcome^42^. Brain tissue is a prominent source of tissue factor^43^ and the location of brain damage is associated with high levels of TF and other procoagulant compounds^44^, which can intensify hypercoagulability and potentially lead to exhaustion and functional impairment of platelets, including their contractile function^45^. Thrombi collected from ischemic stroke patients have a platelet-fibrin network interspersed with cells, and RBCs on average comprise 34% of the volume^46^, which can influence the process of clot contraction^7^. Fibrin clots formed in the blood from ischemic stroke patients have abnormal fibrin ultrastructure that could influence contraction of the fibrin-platelet network^47^. Collectively, these data provide explanations for the recently revealed impaired contraction of clots from the blood of ischemic stroke patients, which displayed a strong correlation with clinical characteristics of the course and outcomes of ischemic stroke^6^. In addition, following clot contraction, the tight seal that is formed around the injured tissue significantly reduces clot or thrombus permeability that is critical to thrombolytic therapy because permeation dictates penetration of plasminogen activators^48^. The effects of local conditions on clot contraction and permeability are poorly understood, yet highly relevant to thrombus growth, stability, susceptibility to embolization, fibrinolysis, and bleeding.

In summary, the data indicate that inflammatory cells, such as activated monocytes, have the potential to enhance clot contraction. This effect can be attributed to the increased TF expression on the monocyte’s surface that produces endogenous thrombin as a powerful source of fibrin formation and platelet activation. The monocyte-modulated contraction of blood clots and thrombi may comprise a novel mechanistic interplay between monocytes and platelets or between inflammation and thrombosis. In view of these findings, the increased monocyte count, their activation and local accumulation that is associated with many (pro)thrombotic conditions may represent a new pathogenic and/or compensatory mechanism that stimulates contraction of blood clots and thrombi. It is possible that the promoting effect on clot contraction is not restricted to activated monocytes, and other TF-expressing cells have this potential as well. However, our findings emphasize the biological significance of monocytes as an important player in thrombosis and hemostasis associated with inflammation.

## Methods and Materials

### Isolation of monocytes

Blood samples were collected into 3.8% sodium citrate (9:1, v/v) from 58 healthy aspirin-free donors following informed consent under approval by the Ethical Committee of the Interregional Clinical Diagnostics Center (Kazan, Russia). All procedures were carried out in accordance with the approved guidelines. Citrated blood was centrifuged at 200 g for 10 minutes, then platelet-rich plasma was removed and phosphate buffered saline (PBS)-EDTA buffer (137 mM NaCl, 2.7 mM KCl, 10 mM Na_2_HPO_4_, 1.8 mM KH_2_PO_4_, 0.1% bovine serum albumin, 2 mM EDTA) was added to the sediment in a volume equal to the removed PRP supernatant. This cell suspension was diluted 1:1 with the PBS-EDTA buffer and layered on top of Ficoll-Paque™ Premium (GE Healthcare Biosciences). After centrifugation at 1,100 g for 20 min at room temperature, the peripheral blood mononuclear cells (PBMCs) were collected, then washed three times in PBS-EDTA buffer (300 g; 10 minutes; 4 °C) and suspended in the same buffer. Monocytes were isolated from PBMCs through the use of Dynabeads Untouched Human Monocytes Kit (ThermoFisher) according the manufacturer’s protocol. Isolated monocytes were washed in the PBS-EDTA buffer (300 g; 10 minutes; 4 °C) and finally re-suspended in PBS without EDTA.

### Characterization of the isolated monocytes

Cell purity was determined using a flow cytometer (FACSCalibur, BD Biosciences) with fluorescently labeled antibodies (BioLegend, San Diego, CA) against CD14 (monocytes) and CD41 (platelets) with isotype controls. Preparations contained 98–100% monocytes of all mononuclear cells and the contamination of platelets varied from 10 to 27 platelets/monocyte (Fig. 3). The cells were utilized within 1–6 hours and the viability was determined to be 91–100% with propidium iodide and trypan blue exclusion assays. Cell counts in blood samples and in monocyte preparations were performed either in a hemocytometer (at 400x) or with an ABX Pentra 60 analyzer (Horiba, Japan).

### Treatment of the monocytes with activators and inhibitors

Monocytes suspended at 1.5 × 10^6^/ml in phosphate buffered saline, pH 7.4, containing 0.1% bovine serum albumin, were activated with 50 ng/ml PMA for 15 minutes at 37 °C and then washed by double centrifugation to remove PMA. To inhibit TF expression via blocking of signaling pathways, a fraction of isolated monocytes was incubated with 250 µM of a peroxisome proliferator-activated receptor α agonist (PPARα agonist or WY-14643)^34^ at 37 °C for 10 minutes prior to PMA activation followed by double centrifugation to remove PPARα (see Fig. S3). PPARα has been shown to play a role in regulation of inflammation and agonists to this receptor have been used therapeutically with marked cardiovascular benefits. PPARα agonists have been shown to influence inflammation and vascular functioning/remodeling^49^ because PPARα activation leads to inhibition of the inflammatory response. The addition of a PPARα agonist results in signaling through the nuclear factor kB pathway which can ultimately suppress tissue factor expression^34, 35^. The TF promoter region contains both activator protein-1 and nuclear factor-kB responsive elements^50^ which allows for PPARα to inhibit the generation and expression of TF. However, the nuclear factor kB pathway has been implicated in a complex crosstalk with other transcription factors such as sTAT3 and p53^51^, and ultimately lead to the enhanced expression/secretion of factors such as monocyte chemotactic protein-1, tumor necrosis factor-alpha, interleukin-1β^52^. Thus, using the PPARα agonist alone does not eliminate the influence of one of these other factors and that was why we coupled these studies with the inhibition of TF with anti-TF antibodies as well as addition of exogenous TF to mimic its effects on activated monocytes.

To block the procoagulant activity of TF, 20 µg/ml of rabbit anti-human TF IgG (Sekisui Diagnostics, Product No. ADG4502) was added to PMA-activated monocytes for 5 minutes at room temperature. Isolated monocytes were re-suspended in phosphate buffered saline, pH 7.4, containing 0.1% bovine serum albumin, at 1.5 × 10^6^/ml and added back to citrated whole blood at a final concentration equal to the initial monocyte count. The average monocyte count in the blood samples was (0.30 ± 0.02) × 10^9^/l. The number of added monocytes was matched to the initial monocyte count, making the total content double the initial monocyte content.

### Assessments of TF expression on the activated monocytes

To quantify the expression of TF (CD142), ~50,000 monocytes in 50 µl of PBS were incubated at +4 °C for 30 min with 5 µl of anti-human CD142 phycoerythrin(PE)-labeled murine antibodies (BD Biosciences) and with 3 µl of anti-human CD14 peridinin-chlorophyll proteins (PerCP)-labeled murine antibodies (BD Biosciences). After incubation with the labeled antibodies, the cells were analyzed using FacsCalibur flow cytometer equipped with BD CellQuest™ software (BD Biosciences). Monocytes were gated based on the size and granularity using Forward Scatter (FSC) and Side Scatter (SSC) channels and 1,000 cells were counted in each sample (Fig. 2C). The results of the TF ELISA (TF ELISA kit, Abcam, Cambridge, MA) show that monocyte activation results in an increase of TF expression from 1.1 ± 0.3 pg/10^6^ cells to 105 ± 5 pg/10^6^ cells at 50 ng/ml PMA and 145 ± 21 pg/10^6^ cells at 100 ng/ml PMA.

### Isolation and characterization of platelets

Platelets were isolated from the same blood samples used to isolate monocytes. Platelets were collected in the void volume after gel-filtration of PRP on Sepharose 2B (GE Healthcare, Sweden) equilibrated with Tyrode’s buffer (4 mM HEPES, 135 mM NaCl, 2.7 mM KCl, 2.4 mM MgCl_2_, 5.6 mM D-glucose, 3.3 mM NaH_2_PO_4_, 0.35 mg/ml bovine serum albumin, pH 7.4). Platelet count was performed in a hemocytometer with a 400× magnification. Platelets were used within 3 hours after blood collection. Cell viability was about 97% based on the maintenance of the mitochondrial membrane potential (Δψ_m_) determined by flow cytometry using a Δψ_m_-sensitive fluorescent dye MitoTracker DeepRed FM (Invitrogen, USA). Non-activated and PMA-activated platelets were added to whole blood in the concentration corresponding to the number of contaminating platelets present in the monocyte preparations to confirm that the PMA-activated platelets were not enhancing contraction induced by PMA-activated monocytes.

Isolated platelets were stimulated with 50 ng/ml PMA for 15 minutes. The results show that PMA indeed activates platelets as judged from PMA-induced expression of P-selectin and active integrin αIIbβ3 capable of binding fibrinogen (Fig. S1). In the presence of PMA, the extent of contraction was also moderately but insignificantly higher (Fig. S2), implying the trend to enhancement of platelet contractile function on top of added thrombin-induced platelet activation. These results justify the necessity of the negative control experiments that we performed with PMA-activated platelets to ensure that the stimulating effect of PMA on clot contraction was due to monocytes, not the contaminating platelets. The lack of stimulating effects of PMA-activated platelets in our control experiments described in the manuscript was because the number of platelets added back to the blood was too small and comprised only about 1–2% of the natural platelet count.

### Clot contraction with addition of TF

Lipidated TF (EXTEM^®^, Rotem) was diluted in 20 mM HEPES buffer, pH 7.4, containing 150 mM NaCl, then added to citrated blood 1 minute prior to addition of thrombin and Ca^2+^ at the final concentrations of 0.625%, 1.25% and 2.5% of the initial commercially available stock solution corresponding to 1.1 pM, 2.5 pM, and 5 pM TF determined by ELISA. A corresponding volume of the diluting buffer was added as a control without TF.

### Kinetics of clot contraction

Kinetics of clot contraction was studied using an original method as previously described^7^. Briefly, blood clotting and contraction were induced by adding final concentrations of 2 mM CaCl_2_ and 1 U/ml human thrombin (Sigma-Aldrich) so that the clot formed within 1 minute. Samples were quickly transferred to plastic cuvettes (12 × 7 × 1 mm) that were pre-lubricated with a residual layer of 4% Triton X-100 to prevent fibrin sticking. The cuvettes were placed into the Thrombodynamics Analyzer System (HemaCore, Russia) and clot size was tracked optically every 15 seconds for 20 minutes. Kinetic curves were analyzed for extent of clot contraction at the end point, average velocity of contraction, and time to reach 5% contraction (lag time). Using a lower thrombin concentration or other clotting triggers, such as TF, without thrombin resulted in prolonged clot formation that allowed erythrocytes to settle, which reduced reproducibility of the assay. With a higher thrombin concentration the clot formed rapidly before the blood sample could be transferred to the cuvette.

### Phase analysis of clot contraction

Rates of the phases of clot contraction were determined using non-linear regression analysis as previously described. ^7^ Clot contraction occurs in three phases: initiation of clot contraction (Phase 1), linear contraction (Phase 2), and mechanical stabilization (Phase 3). The instantaneous first derivative of the kinetic curve was taken to determine local minimum and maximum, which marked the transitions between phases. The Phase 1 and Phase 3 were fitted with an exponential decay equation and Phase 2 was fitted with a linear equation (Fig. 1D).

### Statistical analysis

115 contracted clots were analyzed, with experiments for each condition performed at least in triplicate on cells independently isolated from different donors. Statistics were completed using GraphPad Prism 6.0 using an ANOVA or repeated measures ANOVA followed by Tukey’s test or an exact sum of squares F test. Significance was assessed at a 95% confidence level.

## Electronic supplementary material

Supplementary Information
