# Supplementary material for: Activated Monocytes Enhance Platelet-Driven Contraction of Blood Clots via Tissue Factor Expression
Source: Sci Rep. 2017 Jul 11;7:5149. doi: 10.1038/s41598-017-05601-9 (PMC5506001; doi:10.1038/s41598-017-05601-9)
Supplement: Supplementary file 1 — Supplementary Information [file 41598_2017_5601_MOESM1_ESM.pdf]

Supplementary Information

Activated Monocytes Enhance Platelet-Driven Contraction of Blood Clots via Tissue Factor Expression

Alina D. Peshkova[1], Giang Le Minh [1], Valerie Tutwiler[2,3], Izabella A. Andrianova[1], John W. Weisel[2], Rustem I. Litvinov[2,*]

[1]Institute of Fundamental Medicine and Biology, Kazan Federal University, Kazan 420012, Russian Federation

[2]Department of Cell and Developmental Biology, University of Pennsylvania School of Medicine, Philadelphia, Pennsylvania 19104, USA

[3]School of Biomedical Engineering, Sciences and Health Systems, Drexel University, Philadelphia, Pennsylvania 19104, USA

Supplementary Figures

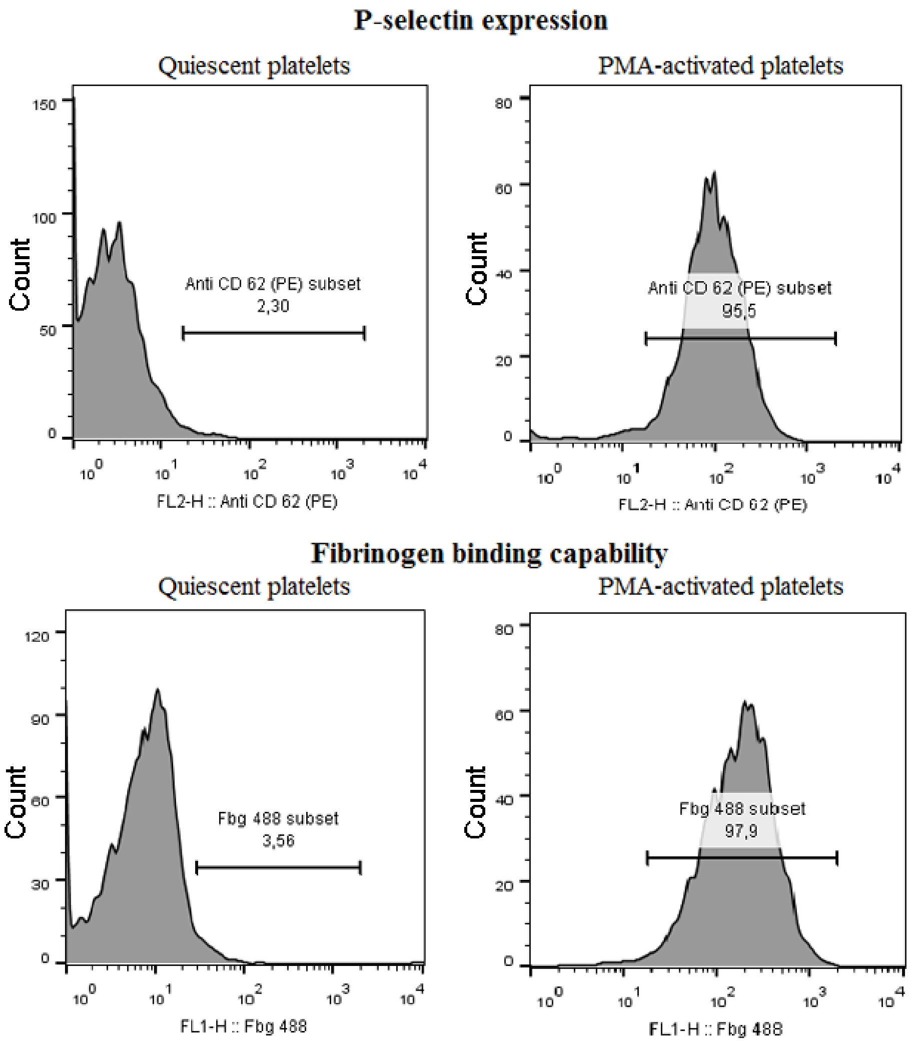

Figure S1. Representative data from flow cytometry of platelets isolated from the blood of a healthy donor before and after PMA-induced activation. The platelets were incubated with either anti-human CD62p phycoerythrin-labeled antibodies (*top*) or Alexa fluor 488-labeled human fibrinogen (*bottom*) before (*left*) and after (*right*) activation with 50 ng/ml PMA. Each plot represents the peak of counts for the fraction of fluorescing platelets. Numbers within each panel (in %) represent the portion of the fluorescing platelets.

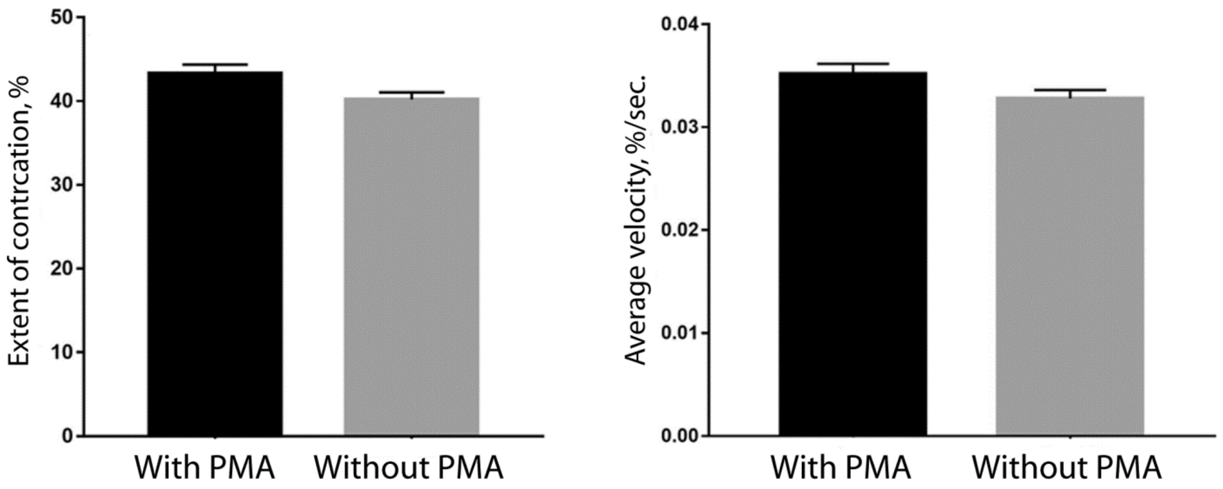

Figure S2. The extent and average velocity of clot contraction in the absence and presence of 50 ng/ml PMA, showing that PMA moderately enhances clot contraction.

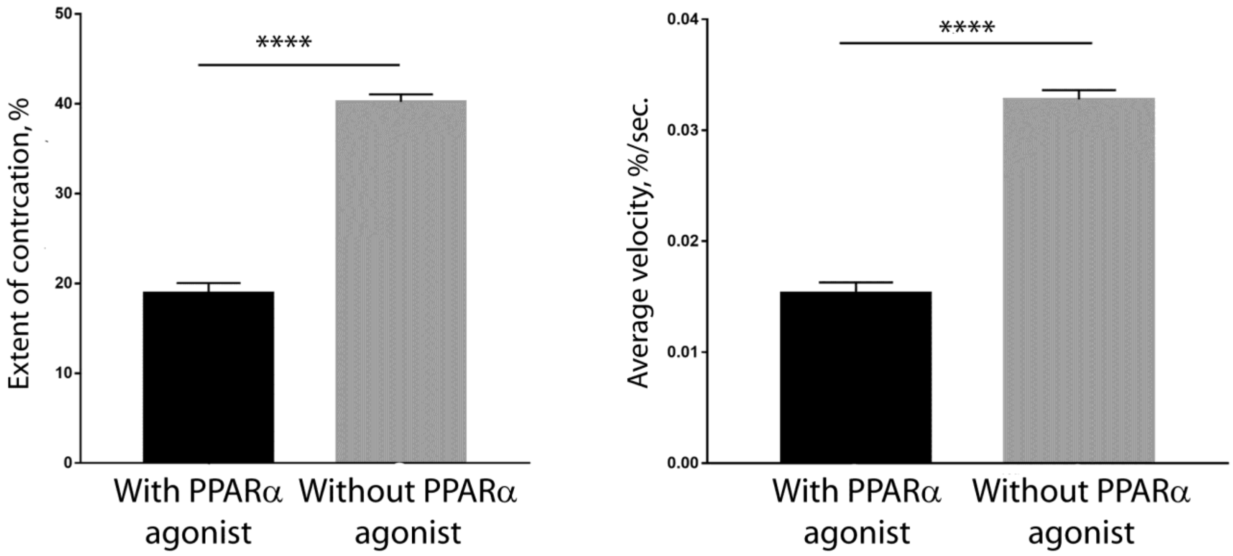

Figure S3. The extent and average velocity of clot contraction in the absence and presence of PPARα agonist. The PPARa agonist (250 µM) added to whole blood inhibited clot contraction by about 50% as expected. First, this result confirms that the PPARa agonist inhibits cell signaling and metabolism not only in monocytes but also in platelets, including their contractile activity. Second, this result justifies the necessity of washing away the PPARa agonist after incubation with monocytes before they were added to the blood sample.

Supplementary Information

Conversion of thrombin activity in units to the molar concentration of thrombin

The conversion of thrombin activity to the molar concentration of thrombin is based on the following calculations.

- We used thrombin from human plasma purchased from Sigma-Aldrich (cat. # T4393) with the specific activity of 1,500-3,500 NIH units/mg protein as indicated in the product specification sheet;
- To convert the NIH units to the International Units (U), we multiplied the NIH units by the factor of 1.15 (1NIH=1.15 IU)[Gaffney, Edgell. The International and "NIH" units for thrombin – how do they compare? Thromb Haemost, 1995, 74(3), 900-903]. Therefore, the specific activity of thrombin in our preparation was 2,250-4,025 U/mg protein (~3,100 U/mg protein on average);
- With 3,100 U/mg protein specific activity 1 U/ml corresponds to 0.32×10^{-3} mg/ml protein;
- Considering α-thrombin molecular weight of 37 kDa, this concentration corresponds to ~8 nM thrombin.
